# “Kids these days pretend to be grownups” *(dekkaedaet):* sexual control and negotiation among young Thai female students

**DOI:** 10.1186/s12889-021-10789-7

**Published:** 2021-05-01

**Authors:** Worawalan Waratworawan, Pimpawun Boonmongkon, Nattharat Samoh, Petcharat Promnart, Thomas E. Guadamuz

**Affiliations:** 1 Center of Excellence in Research on Gender, Sexuality and Health and Department of Society and Health, Faculty of Social Sciences and Humanities, Mahidol University, 999 Putthamonthon 4 Road Salaya, Nakhon Pathom, Thailand; 2 Center of Excellence in Research on Gender, Sexuality and Health, Faculty of Social Sciences and Humanities, Mahidol University, 999 Putthamonthon 4 Road Salaya, Nakhon Pathom, Thailand

**Keywords:** Young Thai women, Sexuality education, Negotiation, Sexual control

## Abstract

**Background:**

Young Thai women are growing up amidst conflicting influences of globalization and traditional Thai culture. They experience confusion about which aspects of their sexuality they can express and must hide. This study examined forms of sexual control and sexual negotiation among young Thai women in secondary and vocational schools.

**Methods:**

Semi-structured interviews and focus group discussions were conducted among 13-to 18-year-old female students attending secondary and vocational schools in six regions of Thailand. Additional semi-structured interviews were conducted with instructors of sex education, school administrators, and parents of students. NVIVO 10 was used to manage, code and assist with data analysis.

**Results:**

Socio-cultural control on young women’s sexualities was found from members of their families, schools, communities, and other students. Young women expressing their sexualities openly through verbal expressions, clothing, and behaviors were deemed negatively and stigmatized as *dekkaedaet* or “kids pretending to be grownups” as it is believed that they are too young and too immature to be responsible for their own decisions on their bodies and sexuality. This dominant discourse subsequently stigmatize and embarrass sexually active women. However, some young women try to negotiate and create options, by utilizing strategies that ensure secrecy.

**Conclusion:**

Comprehensive sexuality education is needed and should be based on the respect for human dignity and sexual and reproductive rights.

## Background

Women’s negotiations for sexual control has long been observed as a mainstream phenomenon since the postmodern era [1]. Sexual control refers to controlling women’s beliefs, decisions and practices about their own bodies and sexuality, including their decision and practices related to their sexual and reproductive health. Women have called for equality as a human being, and for amendments to laws that continue to oppress them under men [2]. One of the key points that have been raised is that women are not oppressed because of biological factors but by socio-cultural ones. In view of cultural feminism, religion also plays a key role in oppressing women and depriving them of their rights [3].

In Asian culture, women are usually framed within family traditions. For example, there are beliefs in several Asian countries that women are their husband’s assets once they are married. For example, young women are valued through their virginity and the fact that their conducts are carried out within the traditions and norms of ideal women [4]. In Thai society, women have been controlled since they were young. Women are not controlled by only men, but by each other as well [5]. Thai culture also plays a key part in suppressing women’s sexual expression through socialization [6].

Nevertheless, women’s movements across the world have been advocating for gender equality and body integrity. People from all walks of life, including students, have also come together to call for equal rights to sexual orientation and gender identity and expression and pointed out the importance of sexual diversity [7]. For example, there have been campaigns that request amendments of marriage laws to include same-sex couples. However, sexual control continues to exist, while be it in more sophisticated forms. For example, sexual control exists in online media, proof that sexual control continues to evolve through different contexts overtime [8]. Yet, there is no absolute form of sexual control that exists without resistance [9].

There have been movements for gender equality and sexual rights in Thailand. People call for rights over their own bodies, clothing, hairstyle and other forms of gender and sexual expressions. For school students, comprehensive sexuality education has been advocated by civil society and multi-lateral organizations [10]. In the past, sex education in school did not exist, but instead was exhibited as sexual control and upholding moral-based values of preserving one’s virginity and blinded loyalty to husbands and fathers, through religion and through general health education. Today, ‘sex’ education is provided in primary schools and high schools, yet still remains under the influence of a ‘traditional’ patriarchal and morally -based sexual culture from the teacher’s point of view and the existing teaching tools/textbooks. Sex education in Thailand is currently a hybrid of the attempts to provide comprehensive curriculum for students under the sociocultural influence of teachers’ abstinence-based attitudes [10, 11].

From the review of the literature, despite attempts to provide students with comprehensive education about gender and sexuality, access to knowledge for young Thai women is still controlled by society, particularly the state, as compared to men [12, 13]. For example, sex education in Thai schools mostly focuses on encouraging abstinence, especially among women [10]. Mass media campaigns and Thai parental socialization tend to reproduce the dominant discourse that young women are not sexual beings, and should focus on being good students and good daughters [10]. Students are controlled by many rules in their school related to hair style, uniforms, dress codes, punishments, etc. [14]. To the best of our knowledge, there is little information that reports on the resistance and negotiation of young Thai women in the context of the current patriarchal dominant discourse. The objective of this study is to examine the various social controls over young Thai girls’ sexual lives, and how they resist and negotiate with these controls. This social phenomenon leads to an understanding of gender construction in Thai society, which is diverse and embodied with values and ideals. A study into these experiences through a postmodern perspective, through which power and construction of sexual identity among young Thai women vary according to the contexts and intensity of power and moralistic surveillance surrounding them, may provide important insights for intervention development.

## Methodology

### Study design

This article provides a view towards sex in Thai society through a postmodern perspective, which believes that there are no universal truths and power cannot be controlled completely [9, 15]. One’s experiences are full of resistance, negotiation and various attempts to establish their identity and their own position in society. This is true for the experiences of Thai female students who face sexual control in diverse and sophisticated forms. They narrate their stories from their experiences of establishing their sexual identity so that they may attain sexual independence in their everyday lives.

This qualitative study was conducted over a period of seven months, between 2015 and 2016, in six regions of Thailand [11]. It is part of a larger research project entitled “Review of Comprehensive Sexuality Education in Thailand”, which aims to understand sexuality education in both secondary schools and vocational schools throughout Thailand. The data collection process for this study was adapted from previous mixed-method studies conducted in schools and communities throughout Thailand [12, 16, 17]. Fifteen focus group discussions of female students (five students per group) were conducted. Another 15 female students were selected from focus group discussion participants to be included in follow-up in-depth interviews. To be eligible, participants had to be assigned female sex at birth, aged 13–18 years old, studying in secondary schools (grades 7–12) or vocational schools (grades 7–9). Furthermore, semi-structured interviews were conducted among 70 instructors of sex education, 30 school administrators, and 30 parents.

The qualitative field guide for focus group discussions (FGDs) of students, and semi-structured interviews for students, teachers, parents, and school administrators can be accessed through a public online depository (available at [11]). Field guides focus on questions related to sexuality education, social meanings of sex and sexuality, and community contexts. Semi-structured interviews tend to be more in-depth on topics of sexual activities, sexual attitudes and sexual negotiations than FGDs. This is because interviews are conducted one-on-one versus FGDs where participants are providing information surrounded by their peers. Among teachers, questions revolve around values and attitudes towards sexual matters, while among parents, questions tend to be related to the contexts and situations of students’ sexuality and sexual matters in the family. Among school administrators, questions tend to be concerned with policy and sexuality education curriculum.

The study process started with encouraging students to draw a picture of their sex education class and its activities, according to their own perceptions and personal beliefs, and then having each student explain the drawing to the group. Researcher then use the drawings to probe other questions like “what is sexuality education in your view?” … “what do you think about having a steady or casual sex partner while still a student? … “what do you think about teen pregnancy?” … “what do you think about abortion?” (more details available online at [11]). This methodology encourages students to feel more comfortable, to take their time in pondering upon their own sexuality, and share significant stories from their lives with the researcher and their peers. Drawings were also used by the research team to conduct content analysis of sexuality issues in schools [18]. All drawings presented in this article were photographed with verbal permissions from study participants.

### Recruitment of research participants

Recruitment of participants have been described elsewhere [15]. Briefly, participants were recruited from five schools in each province (totaling 30 schools across all six provinces), four general schools and one vocational school, using multi-stage cluster sampling. To do this, one classroom was randomly selected for each grade, then six students were randomly selected from each classroom using their student identification numbers. To avoid potential stigmatization, we did not recruit LGBTQ students directly, but rather relied on their self-identification or through their drawings (e.g. same-sex relationships) and their explanations of their drawings during FGDs,

One to two instructors with the most experience in sex education were recruited from each school for a semi-structured interview. In the recruitment of students’ parents, the coordinating teacher assisted the researcher with the selection process.

### Informed consent/assent and proxy permission

Mahidol University Institutional Review Board (MUIRB) reviewed and approved this study. Participants 18 years and older provided written informed consents, and participants 13 to 17 years provided written informed assents. For participants who are younger than 18 years, additional written permission was obtained from either guardians or from homeroom teachers who acted as adult proxies. This additional process was informed by our previous study [19], reviewed and approved by the MUIRB. All participants were given a participant information sheet as well as a consent form (and assent form where appropriate) that provided details of the study, including a permission to audio record the interviews/focus group discussions. Students’ participation in this study was voluntary and anonymous. Researchers used alias/codes for student’s names and school’s names, when reporting findings. Focus group discussions and interviews were conducted in closed and isolated rooms. Safe zones (no teachers or administrators in the room) were created to conduct students’ interviews and confidentiality and privacy were emphasized. In FGDs, facilitators avoided focusing on sensitive issues that could lead to stigmatizing or blaming of students such as personal experiences of abortion. However, we introduced questions that are related with other students’ life narratives or their opinions on public issues. Personal narratives which may lead to stigmatization were instead asked during one-on-one in-depth interviews. Each student received a souvenir (valued at about 100 Baht or 3 USD) for their participation in the focus group discussions and in-depth interviews.

### Data analysis

Six researchers assisted with data collection who also took part in coding and analyzing the textual data using thematic analysis with the help of Nvivo 10 software. We organized and classified our data into themes and sub themes so that we can properly address the research objective. The coding process includes: a) all interviews were audio taped, transcribed and checked by the research team, b) the research team read the transcripts and coded the information by themes, c) coded themes were then verified among the research team members, and then d) themes were organized and grouped into sub themes in order to address the research objective. See Table 1 below for details on themes and sub-themes.

**Table 1:** Summary of key themes, sub themes and explanations

Themes	Sub themes	Explanations for each theme
Attitudes towards sexuality	• Abstinence • Pregnancy • Sexual rights	Students’, teachers’, and parents’ attitudes towards sexual relations among teenagers
Practice of sexuality	• Sexual practice viewed by family • Sexual practice viewed by teachers • Sexual practice viewed by students • Sexual practice viewed by community /society/media	Sexual practices including controlling, monitoring, negotiating, opposing against all matters related to sexual relations in teenagers
Sex education	• Attitudes towards teaching sex education in schools • Knowledge of sexuality • Access to knowledge of sexuality • Application of knowledge of sexuality	Contexts of sex education in schools, knowledge of sexuality, access to knowledge of sexuality and application of knowledge of sexuality.

We also triangulated the data among students, teachers, administrators and parents in order to confirm and validate the findings, and to reduce bias among researchers. To do this, we confirm findings through asking similar questions among students, teachers, parents, and school administrators. We also involved a team of six researchers to collect, code and analyse data to minimize bias among researchers.

Further, we used Foucauldian conceptualizations [9, 20] that power is present and can be exercised in different, sophisticated forms. It is exercised directly towards the human body and can transform one’s body into a docile body, under the influence of social construction of truths. Power is also used as a repressive means to inspect, monitor and repeatedly survey people [20]. But at the same time, individuals also show attempts to build their identities through resistance, negotiation and technologies of the self [21, 22]. Likewise, young Thai women seek resistance and negotiation in order to establish their sexual identities through different forms of social surveillance, which are reproduced and normalized through social discourse such as being a good student and daughter. This information will be discussed more in the results section.

## Results

### Forms of control and surveillance of young girls’ sexuality

This study examines different forms of surveillance and control, which is passed down through Thai social structures. Different institutions also have a significant part in forming sexual control over young female students, such as family, community, religion, school, as well as friends, who take the role in monitoring other students’ sexual behaviors, supporting their punishment, and stigmatizing other female friends whose conducts are in conflict with Thai society and culture.

### Parents: “preaching” and “monitoring”

“*Parents are the first teachers in life.”* This is the classic sentence that resonates in Thai parents’ minds to make sure they teach their children to be good persons. In Thai society, parents have a key responsibility for teaching their children and are entitled to punish them if they do not conform to social rules. This conformity becomes more intensified, especially with daughters. Parents often control their daughters’ sexual behaviors by instigating fear, setting high expectations, and claiming a sense of gratitude. The intensity of Thai parents’ sexual control over their daughters varies from preventing them from dating to preventing them from even going out one-on-one with a male friend and preaching about the ills of teenage pregnancy or pregnancy before marriage.

“*I try to observe my daughter to see if she uses her mobile [phone] a lot… I am more concerned about her because she is a woman… But if one day I find out that my daughter has a condom in her bag, I will be quite worried.”*

*--*A mother of a female student from a secondary school (grade 9)

*“I taught her. I asked her to think thoroughly … Don’t be too curious about this and that (sexual relations)...There are several bad examples you can see in the news and in reality. These things [sex] can make them lose their future.”*

--A father of a female student from a vocational school (year 2)

*“My mother said that if I have sex with my boyfriend and accidentally become pregnant, she will not accept me as her child anymore. And, she won’t allow me to stay in her house anymore and I have to move out.”*

--FGD, a female student from a secondary school (grade 7)

Most Thai parents will usually tell their daughters that having an unplanned pregnancy is unacceptable. This is considered to be an act of ingratitude towards parents. Not only their daughters, but the parents themselves, will be condemned by society for their failure to remain within the norms of society. However, some families still give their daughters the right to have a boyfriend.

*“My mother often says she allows me to have a boyfriend, but she begs me to not go anywhere privately. If my boyfriend wants to meet me, have him come and talk to me at home. She does not allow me to go anywhere else with him, so to prevent others in society from gossiping. And so my boyfriend is used to seeing me at home while my mother chaperons. My mother said that if she stays home, my boyfriend can see me at home. If my mother isn’t at home, my boyfriend won’t be allowed to come. Even though the two of us held hands at home, our mother didn't scold us. My mother has seen us being close with each other.”*

-- FGD, a female student from a secondary school (grade 8)

In the above example, the mother allows her daughter to escape the societal controls and constant surveillance of Thai society by providing a safe haven at home. However, the mother still controls and keep a ‘watch out’ for her daughter and her daughter’s boyfriend, establishing rules to what is allowable behaviors (e.g., no sexual intimacy).

### Religion and spiritual control

Most students perceive pregnancy while in school as unplanned and a mistake. If a pregnant girl chooses to “keep the baby,” for example, they are considered to be doing a good deed and deserve forgiveness for their ‘mistake.’ However, if a pregnant girl chooses to abort or “take out the baby,” they are considered to be evil, doing a bad deed, not good person, and should not be forgiven for their ‘mistake.’ They are breaking the first precept of Buddhism, which is about abstention from killing all living beings. Furthermore, abortion is illegal in Thailand and so regular clinics and hospitals will not offer this service. Instead, students seeking abortion can do so safely through non-governmental organizations and their networks of clinics and hospitals, illegally.

*“Abortion is a sin and they’ll think you have a bad soul.”*

-- FGD, a female student from a vocational school (year 3)

**Fig. 1:**
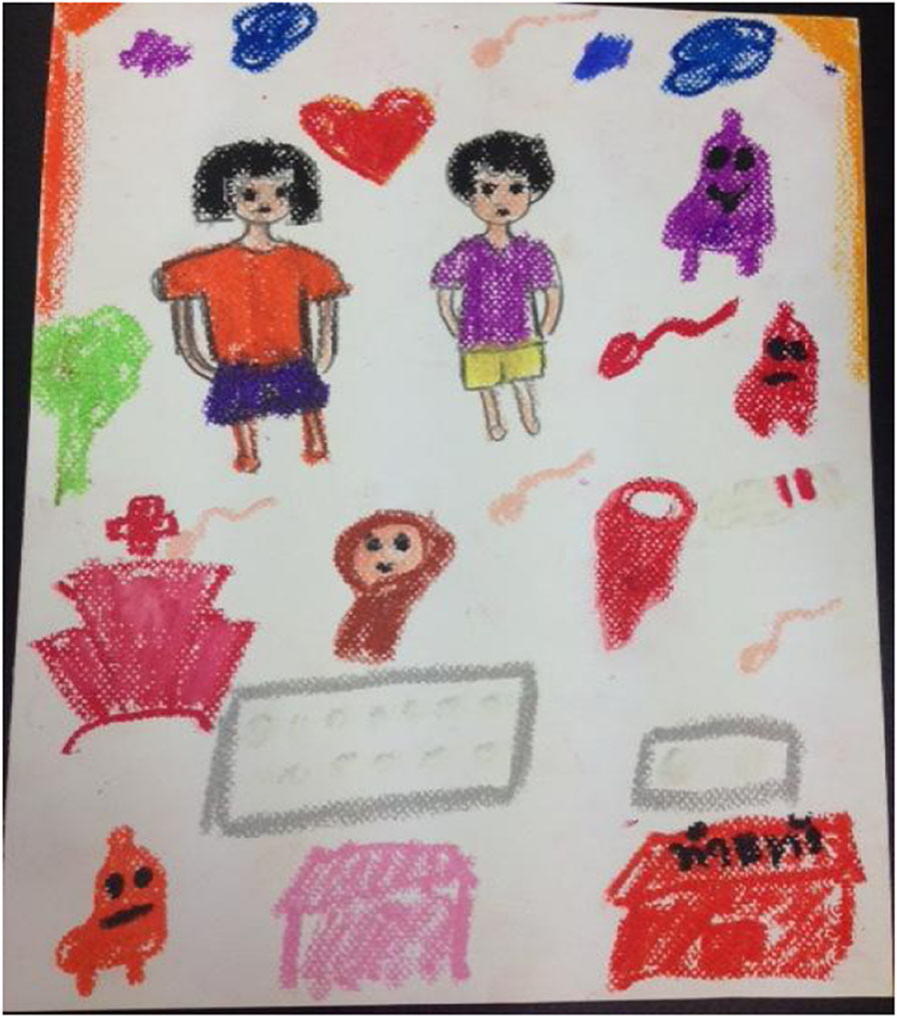
Picture painted with dark red color, which suggests an abortion, aborted baby, and sad baby ghost due to being aborted. (Fig. 1 does not violate copyright; permission has been obtained from the student of the FGD)

Many students also believe that the spirit of the aborted child will come back and take revenge on the mother (Fig. 1). Aside from the person who aborts the baby, individuals who assist in providing advice, assist in taking the student to an abortion clinic and those who are providing abortion services are all taking part in bad deeds and will be punished. Therefore, most students try to refrain from going to their teachers and friends for advice on abortion because they do not want their teachers and friends to be considered bad persons or doing bad deeds.

Some female students said that having a sexual relationship while in school is breaking the third precept, which is abstention from sexual misconduct. The third precept in Buddhism claims that one shall not have sexual relations with other people’s life partners. A female student stated that “children belong to their parents.” Having sex while a minor (children under 20 years old, still in school, no job, and cannot financially support themselves) is sinful since children belong to parents and parental permission is needed, else one is doing a very bad deed. As stated earlier, in Buddhism, parents are birth givers and can be compared to, or considered to be, deities of their children. Therefore, children cannot do bad deeds to their parents as it is compared to doing bad deeds to deities, which is very sinful.

However, some students think that abortion is a personal right. Especially in the case that the boyfriend is not responsible, a woman should have an abortion. These students feel that abortion should be legalized in Thailand so that safe abortion practices can be provided.

*“Abortion is our rights. We can decide whether to have an abortion or not. If our boyfriend rejects responsibility for our pregnancy, we must have the right to have an abortion. We may only be sad for a while. But if we undergo an illegal abortion, it's not safe because the surgical instruments may not be clean, and it may cause infections. If we have a legal abortion, we will be safer. I would like to give you an example of a popular drama series called “Hormones” which correctly presented teenagers’ everyday life and the struggles they go through. One episode dealt with an illegal abortion that was done in secret, without telling her parents and where she experienced unstoppable bleeding. This movie teaches us a lot about sex.”*

-- FGD, a female student from a secondary school (grade 7)

### Schools teach and punish

School is where students spend most time during the day—usually with their teachers and schoolmates. It is the place where structures, rules and penalties are formally set up. It is also the first place where teens spend time together. Schools are responsible for teaching and providing students with an education. Therefore, there are different rules and regulations set up for controlling them to make sure that they are well-equipped with skills and are “good” until they graduate.

**Fig. 2:**
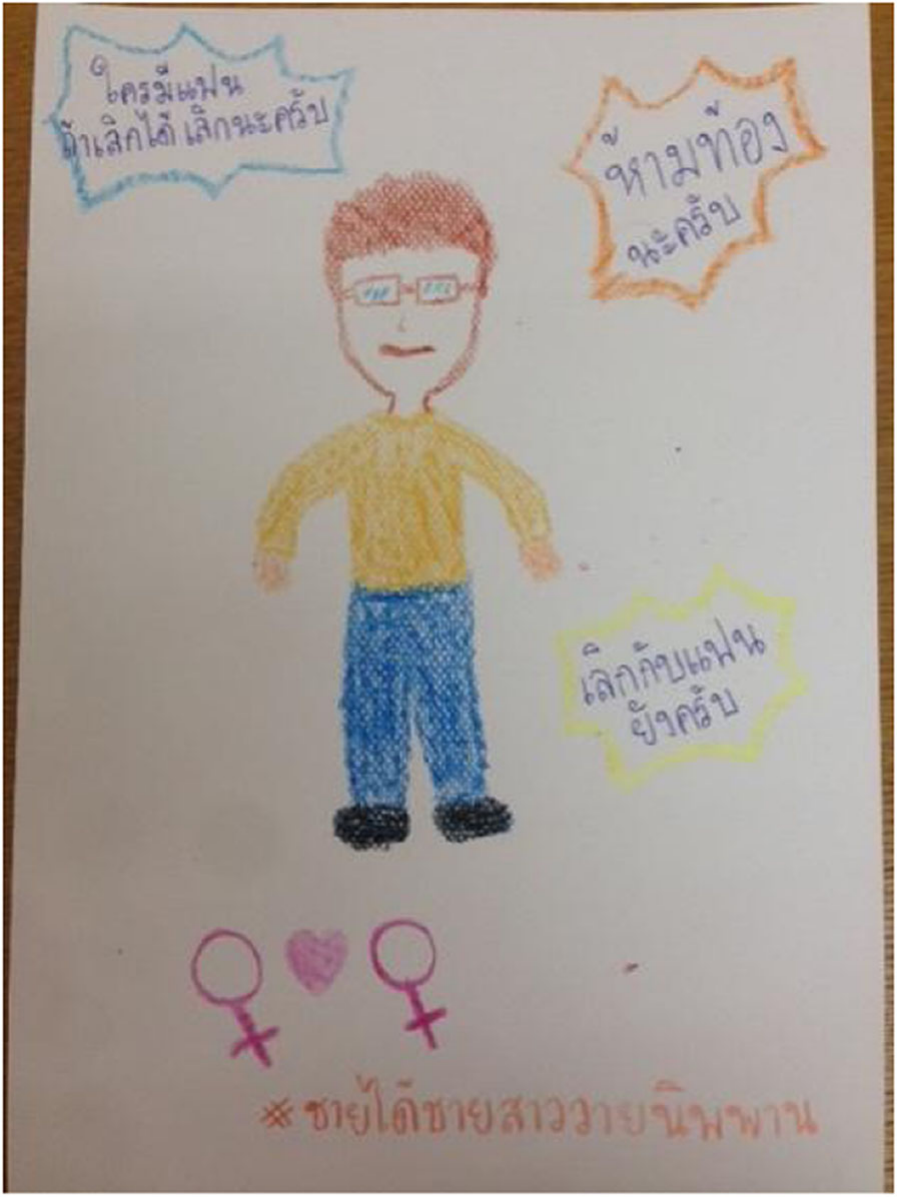
A picture of a teacher who teaches students every morning the following admonitions: “*For anyone who is dating, if possible, end it,” “please don’t get pregnant,” and “did you break up with him yet?”* (Fig. 2 does not violate copyright; permission has been obtained from the student of the FGD)

Society believes that students must be strictly controlled to make sure that they are moving forward in the right direction. Additionally, any diversity among them should be eliminated to make it easier for schools to control them. For example, sex topics are forbidden in schools because “*student’s attention to sex topics will make their school grades drop.”* While pregnancy in school is certainly considered an obstacle to students’ learning, the most important impact of pregnancy while in school is that it could affect the school’s image and credibility in the community and by the Ministry of Education. Student pregnancy rates become an indicator of teachers’ failure to teach and take care of their students. These examples of control were repeated from several students across two FGDs: one school has a rule that female and male students are not allowed to sit side by side anywhere within the school property, and other schools have a rule that dating between the opposite sex is not allowed. In some schools, teachers will lecture to their female students everyday about “waiting” and abstaining from sex. Many schools instigate fear on their students, that they will be condemned if they are dating and then later become pregnant while in school (Fig. 2).

*“I give them some bad examples of female students who became pregnant while in school; they are forced to leave school. Some of them did not continue their study [even after the pregnancy]. They had to work in sugarcane plantations or rice farms, which is more exhausting than studying...”*

A teacher from a secondary school

*“There are some students who love each other. When teachers find out, they reduce the students’ behavioral scores. If the scores get too low, they will have to attend a military camp in order to readjust their attitudes and behavior.”*

-- FGD, a female student from a secondary school (grade 8)

In many Thai public schools, students are given a set score for their good behaviors at the beginning of the year. Then for the rest of the year, this score may be reduced overtime as punishments, and if the minimum threshold is surpassed, then the student is expelled immediately from that school.

Moreover, some schools establish a group called “White Spy,” (but most schools call them “Inspector Student” or “Class Representative” who take the same role of spying other students and reporting to the administration), which is conducted by students with “good behaviors”—in the eyes of their teachers. This group will be responsible for monitoring the sexual behaviors of their peers both inside and outside of school, including online spaces such as Facebook and Line chat groups. For example, they will report to their teachers when they find their peers change their relationship status on Facebook, uploading a ‘couple’ photo or any ‘sexy’ photos, which are not allowed. Any female student who is reported will face different levels of punishment: Level 1—teachers will call them and give a warning; Level 2—teachers will reduce their behavioral scores (each student begins with 100 and once scores fall below a certain level, students are expelled from the school); and Level 3—teachers will ask their parents to come to school.

*“It is 100% certain that they [students] have porns [pornography] in their mobile phones. They even make their own clip videos. Sometimes the way they express themselves makes them look like a stripper... I have spies who are good students. They will dig some information about those [bad] students for us.”*

A deputy director from a secondary school

Apart from penalties as reducing their behavioral scores and talking with their parents, teachers choose to punish students by condemning them in front of everyone, which is aimed to show as examples to other students that this student is a bad example and do not follow her/be like her. In every Thai school, all students will gather each morning in front of the flagpole for the national anthem. After that, teachers will provide them with school updates, preach about life and importance of studying. In some schools, teachers choose to punish students in this space, in front of everyone. Sometimes students are spanked in front of everyone as a “bad” example for all to witness. These types of stigma created and sustained by school administrators and teachers also contribute to the high drop-out rates of pregnant students, especially in big cities. Although by law, pregnant students are allowed to continue their studies in schools, but in reality, pregnant students receive tremendous social pressures and negative remarks from school administrators, teachers and even by their peers, which subsequently forces them to drop out of school.

*“...Here is what I usually suggest to pregnant students: I think you should drop out of school for now and come back to study again after you give birth to your baby. But I suggest you choose to study in a vocational school because you already have a family and you need to work to support your family”*

A deputy director from a secondary school

Despite controlling young Thai girls’ sexual life, some schools, including teachers and administrators, see the importance of providing children with sex education, understanding and advice on sex-related matters without accusing and giving them pressure.

*“Sex education is included in all subjects and activities at our school. Teachers who teach sex education to students should have an open-minded attitude, understanding of rights and equality, good teaching techniques, acceptance of diversity, being funny and generous, acting like father or counselor, and being able to give proper guidelines when they have problems. Most importantly, the teacher must be able to keep their students’ secrets.”*

A director from a secondary school

*“There are two aspects of teaching students about sex. We have to teach the students every aspect. We must allow them to share their ideas, encourage them to critically think and make decisions by themselves ...”*

A teacher from a vocational school

### Peer and senior students: from friendships and support to reproducers of societal control and surveillance

A senior student commented on some of her junior peers that she does not like them because they sexually express themselves too much by the way they dress, speak, and act. “*Kids nowadays often pretend to act like grownups.”* These kids are considered to be part of vulnerable groups. They are a group of students that good students (teacher’s perspective) do not like and do not usually hang out with. “*They always refer to the man they are dating as their pua [lover/husband] …*. *I just don’t like the way they show off like that.”*

*“Nowadays, there are a lot of “dekkaedaet” (kids pretending to be grownups) … They would go to a bar near the school and pretend to be grownups.”*

FGD, a female student from a vocational school (year 3)

Some good students (teacher’s perspective) usually regard sex topics as something “dirty,” and teens should not think and talk about it, as it is just not the right time for them to know. This attitude blocks them from accessing useful information to make an informed decision about sexual issues for themselves in the future.

*“When teachers teach about sex, they teach boys how to use condoms … , but the girls don’t want to watch. We feel that we don't want to see such training, so we turned our back to it and closed our eyes. We just don't want to pay attention to it. The teacher showed us many pictures. They are ugly and terrible pictures.”*

-- FGD, a female student from a secondary school (grade 8)

Moreover, teachers tend to think that knowledge about sexual relations is only for grownups, and students should know only when they can start earning a living by themselves or after they get married. Sexual relations should only occur once they are socially and economically prepared, or once they receive permission from their parents.

*“My mother said that every woman can be a good woman but being a good woman for our family means being virtuous before having sex.”*

-- FGD, a female student from a secondary school (grade 9)

On the contrary, for the young girls who pretend to be grownups, as mentioned earlier, they have the opposite view. They think it is their right to have a boyfriend or to have sex. In this way, they are not *dekkaedaet* because they are not pretending and one may argue that they have already grown up.

*“They call us “dekkaedaet”, but I know what I am doing. I just didn't trouble anyone. I prevent myself from getting pregnant."*

-- FGD, a female student from a secondary school (grade 10)

### Forms of negotiation among young Thai women

Various institutions—from family, school, religion, community to groups of friends and senior students—play important and unique roles in the surveillance of sexual behaviors of female students. As a result, it makes them feel “*uncomfortable”* and “*repressed.”* They, however, respond by negotiating for their sexual independence.

### Negotiation through conversations with parents

With pressures from being constantly monitored, female students choose to hide from their parents and teachers the fact that they are dating a man or having sex with him. When talking to their parents, they choose topics like school and friends, but never mention love or relationships. They will not show curiosity or ask their parents about topics related to sexual relations or sexuality. They would rather use the word “friend” to refer to everyone, including their boyfriends, so that parents do not ask more questions or feel worried.

*“I just told my mother that I went with my friends. But I knew that she knew I went with someone special...she does not believe that I will have sex with him though...I didn’t tell her that I have a boyfriend and that I am really prepared for sex.”*

IDI girl student *secondary school* (grade 11)

They will also pretend to obey their parents’ teachings about sex and sexuality and keep their sexual relationships on the down low. They will not argue when their parents teach them or remind them of the importance of chastity or remind them the meanings of “bad kids.” Their parents will usually find an opportunity to teach and talk about sex topics while having dinner or watching TV together with them. They will pick up some bad examples on unplanned teen pregnancy and sexual relations from the news on TV, from soap operas/dramas, and/or from neighbors’ stories and gossips to teach their children. The children will act as if they agree with the parents so that they will not worry. Some kids may even comment (in agreement) on these teachings and stories or may even find examples themselves. Acting this way can help set them free from their parents’ control.

If their parents find out that they are dating someone, they may exsert more control over them. For example, parents may start specifying time for their kids to arrive home from school, stopping them from going out one-on-one with all male friends, personally accompanying their children to and from school to prevent them from going anywhere with a man, and forcing them to break up with their boyfriends. With these pressures, some female students choose to avoid facing any problems with their parents by keeping the truths secret from them. In this way, students neither violate their parents’ teachings or conform to them.

### Negotiation for sexual independence

Young Thai women continue to negotiate with societal expectations in attempt to fight for their womanhood. To be a good daughter, for example, one has to be obedient to their parents, teachers, and to the social norms of the larger Thai society. Students put forth strong efforts to finish their schooling, while preventing themselves from unplanned pregnancy while in school. These efforts also reflect their pursuit of sexual independence. They are also fully aware that they have to be responsible for their own bodies by protecting themselves from unplanned pregnancy with the help from various sources of information such as the internet or their friends.

“*...I take an emergency contraceptive pill every time when we have sex. I am afraid of being pregnant...We have sex once a week. It is safer to take the pill. I take one pill before having sex and take another pill right after having sex.”*

IDI, a female student from a secondary school (grade 7)

With all the aforementioned reasons, pregnancy while in school has become female students’ biggest concern. All the secrets they have kept away from their parents (e.g., dating and having a sexual relationship with a man) will be exposed, causing them to lose freedom in school life and putting their romantic relationship at risk. Their parents will be disappointed since they may have to drop out of school to do parenting at home, become stigmatized as a ‘bad kid,’ and be condemned by Thai society.

Therefore, female students would try every way, regardless of potential risk and harms that may come, to enable themselves to continue having a sexual relationship without getting pregnant. Most of them will either seek information from the internet or ask for advice from their close friends. Students usually seek information on pornography, contraception, pregnancy and abortion. Some of them choose to take emergency contraceptive pill every time they have sex; others choose to take birth control pills. However, some find it difficult to hide birth control pills from their parents. Some do not want to carry condoms in their bags because they fear of being caught and stigmatized as a ‘bad’ woman by other people, including their boyfriend. So they choose to ask their partner to bring and use a condom instead. If their partner refuses to use it, then they will have to take the emergency contraceptive pill to prevent themselves from getting pregnant.

However, according to the data, some female students have to take emergency contraceptive pills almost every time because their boyfriend always refuses to use condoms. Their boyfriend will usually claim that wearing a condom is not fun and makes them feel strange during sex. This makes the female students feel that it is their responsibility to prevent themselves from being pregnant in order to maintain their independent lifestyle. Furthermore, most female students choose to ask their boyfriends to buy emergency contraceptive pills for them after having sex.

### Negotiation in online spaces

Female students’ sexual expressions are not only controlled in offline spaces but also in online spaces. Teachers, “White Spy” students and parents will always monitor their behaviours through online channels. So, they choose to avoid being controlled in online space by creating the other Facebook account. They will mainly use their original Facebook account for communicating with friends, teachers, parents, and others, including their teacher’s “White Spy.” Through this Facebook account, they will upload general content, and nothing related to sex and sexuality. The other Facebook account, however, is used for communicating with close friends and their boyfriend only. Through this account, they will upload their daily activities, openly update their relationship status (e.g., “In a relationship”), and even upload their couple photos. They use two Facebook accounts as an approach to escape the policing by their parents and teachers, and to avoid being punished according to school rules. If their teachers notice that they have a boyfriend or put “In a relationship” status or uploading couple photos, they will likely have their behavioral scores cut, being condemned, made as a bad example in front of every other student, or being asked to have parents come to school.

“*I created two Facebook accounts. One account is for them (teachers, white spies, parents, and relatives) to stalk, and the other is only for communicating with my close friends and boyfriend.”*

FGD, a female student from a secondary school (grade 8)

In addition, most female students choose to seek information about sexuality e.g. birth control pills and pregnancy by themselves from the Internet. They choose to not seek detailed advice from their teachers or parents because they are afraid of being judged. Moreover, the information they receive from the Internet is often more detailed than that they receive from their teachers e.g. information on fertility awareness methods.

“*My teacher taught me about fertility awareness methods. But when I asked her some questions, she gave me unclear answers. So, I searched for the information by myself from the Internet. Now I have a clear understanding of it”.*

FGD, a female student from a secondary school (grade 7)

## Discussions

### Multi-dimensional control and self-surveillance

Social structures in Thai society have together reinforced and exerted a kind of sexual control over young Thai women [23]. For example, women are divided into two groups: good women and bad women [24]. They live their lives by strictly adhering to this binary concept and choose to walk on the path laid down by society. Different discourses enhanced by specific Thai cultural contexts have been subtly absorbed into their consciousness (e.g., discourse on gratitude). In Thai society, there is a belief that children are their parents’ possessions. It is important for children to obey parents and repay them with kindness [25]. This belief has led young women to lose power over their bodies since they always have to rely on their families in making any sexual decisions for them. In addition, young women also derive ideas of monogamy from religious teachings imported from the West [26], which has been repeatedly reproduced through popular media and textbooks [11].

Young women have been controlled, trained, and taught to recognize the value of their virginity [9]. Young Thai women thus believe virginity is critical [27]. The idea of only having sex after marriage has been cultivated since they were young. Aside from negative attitudes towards having sex while in school, young Thai women also have fantasies about romance and a good husband [28]. These dreams are shaped by the Thai male-dominated society [29]. Thai women, for example, may feel guilty if they have sex before marriage, which violates social expectations and social scripts. Having first sexual relation with someone before marriage usually makes young Thai women feel guilty, confused and insecure. Their guilt may even become intensified when they learn that the man they had sex with is not a good man or someone they deserve [29].

Social structures at all levels have played a key role in exercising biopower over young Thai women [20], which forces them to be obedient and submissive to the dominant discourse. They are constantly controlled by society, even in their dreams. Men want to make sure that women follow their teachings, rules, and traditional practices determined or expected by society. If they fail to follow these rules, such as having sex before marriage, having sex with someone other than their husband, or having sex with more than one man, it will make them feel guilty and not a good woman. For example, if they become pregnant while in school, not only will they know they will be punished, stigmatized, and condemned by society; they will also feel that they deserve to be punished. Additionally, they will also stigmatize themselves as an undesirable person. They will feel guilty, ashamed, and find it difficult to live happily in their everyday lives [30]. These situations also make them stressful, and to cherish their virginity. This phenomenon then reproduces sexual control over and over to future generations.

The exercise of biopower is therefore so sophisticated that young women do not feel that they are being controlled [9]. This kind of power is clearly exercised by authorities such as teachers, parents and the general moralistic values of society. On the other hand, biopower also triggers self-surveillance among young Thai women, who try to resist any sexual controls over them. Ironically, while in quest of their sexual independence, both in offline and online spaces, they still have to conceal some truths and continue conforming to the social expectations under the influence of the reproduced male-dominated discourse e.g. “do whatever to not get pregnant,” “do whatever but complete your study on time,” etc.

It is remarkable that students in Thailand face such control over their sexuality, as indicated by our findings. Thailand is widely known for its openness of gender and sexuality diversity and expression, whether it be through the openness of sex work and sex markets, the proliferation of gender and sexual identities, the frank discussion of sex and sexuality in social media, and even representations of homosexuality and transgender people [15, 17, 31, 32]. Despite all of this, Thai schools and Thai parents are extremely conservative when it comes to sex education for young people.

### Negotiating sexuality and sexual and reproductive health

The importance of comprehensive sexuality education in Thai contexts is still overlooked, especially topics on sexual rights, gender, power, and negotiation. Most female students, for example, still lack a general understanding about their sexual rights. Although they are taught about sex education in school, they do not necessarily have access to practical information about sexuality that they can actually apply to real life [11]. That is why they are forced to seek information about sex and sexuality by themselves from the Internet [33].

The Internet has become safe spaces for young Thai women seeking knowledge and seeking a place to express their sexuality. What is interesting and different about Thai social context is that parents, teachers and society invade these online spaces in order to conduct monitoring and surveillance of young Thai women’s behaviors. At the same time, these women also use online spaces to resist, negotiate and monitor others too. Online spaces, therefore, become a playground for operations of discourse as well as spaces for escaping, negotiating, and developing their gender identities that may or may not strictly conforms social expectations.

Their identities are therefore an embodiment of the contestation of sexual independence and of conformity to social expectations. Through a technology of self, which is part of their resistance, these young women redefine this identity as self-awareness [20]. With this new meaning of their newfound identities, they can grow, learn and become subjects in the sexuality contexts in Thai society. These young women, who are commonly referred to as *dekkaedaet*, are remarkably resilient and courageous. Future studies should examine the strategies and mechanisms related to resilience among *dekkaedaet,* who despite having to resist and negotiate with the dominant discourse, still turn out to be happy, healthy and sexual.

Based on several studies [12, 15] educating students about sex by means of threatening, scaring, and forbidding them simply do not work in terms of sexual health promotion, or reduction of unwanted teenage pregnancies. Sex education based on scare tactics has also been counterproductive [11]. Students are taught that sex is disgusting, is shameful, and should not be discussed while they are in schools. Their roles, as dictated and scripted by society, is to be good students and good daughters, not sexual beings. Sexual pleasure is therefore not meant for women, but for men and boys—sex thus becomes an activity for pleasing husbands and for reproduction [11].

In fact, comprehensive sexuality education where honest discussions are encouraged can significantly reduce unplanned pregnancy rates among young women [34]. Yet, the Thai government still does not allow for comprehensive sexuality education to be included in Thai schools, as part of the sex education curriculum [31]. However, young Thai women still show their attempts to not accept this predominant dogma, and instead navigate and negotiate with society and its social structures and pressures. These individuals choose to maintain their sexual independence and perform as a “good girl” as expected by society (e.g., having two Facebook accounts), while fulfilling their sexual desires (e.g., having a boyfriend and being sexually active). They may try different ways to prevent themselves from being pregnant like taking contraceptives or by negotiating with their partners to use condoms, all while staying in school.

## Conclusion

Given the prevailing gender norms, young women remain at risk of problems related to sexual and reproductive health (e.g., from frequently taking emergency contraception pills to contracting sexually transmitted infections, or having unplanned pregnancy and unsafe abortions [35]). It therefore suffices to conclude that these risks where young Thai women are exposed to are the result of closed-minded attitudes and beliefs embodied by the socio-cultural structures of Thailand. Young Thai women are taught to be fearful of embarrassing their parents, of being ungrateful daughters, and of being gossiped and ridiculed by society in general.

This paper reveals young Thai women’s attempts to negotiate and express their sexual identity and sexuality through their own spaces, especially online spaces, which are new and safe for them. They also try to employ their womanhood and youthhood to fight with social operations that come along with social expectations and other rules in both offline and online spaces. Therefore, it is necessary for Thai society or any organizations or institutions under it to provide opportunities for them, understand their body rights, recognize their needs for an expression of their identity as a human, and their sexual rights, which are basic human rights. They should also be provided with a safe zone where they can speak openly and freely, to seek advice, to exchange information, to learn, and to make their own decisions about sexuality and sexual health in their everyday lives.

### Recommendations

It is important for the family, society, and schools to offer young Thai women with effective sexuality education, which is not limited to only preaching about chastity. Instead, providing students with comprehensive sexuality education should be a main focus, which includes giving them choices and education about sexual rights. It is also necessary to equip them with critical thinking skills to encourage them to analyze opinions, beliefs, myths, as well as any forms of reproduced cultures linked to gender bias, views on women as objects, and suppression of women’s rights passed down through societal structures. Sexual topics should not be regarded as taboo. Building a safe zone with supportive environments for students to openly talk about sex, sexual rights, power, and sexual pleasure is urgently needed. Our findings clearly show that young women’s sexuality was influenced by society at multiple levels. Social media has become an ever more important space to express their identities, which may provide a turning point for society to understand and ultimately compassionate to their struggles for the same sexual rights, and the right to express their gender and sexuality. We suggest that future studies should focus on the impact of social media on young women’s sexuality.

### Limitations

Since this is a qualitative study, our findings are not generalizable to all students in Thailand. Second, we did not assess the religion of study participants and so we cannot infer the relationships between religious aspects and sexuality/sexual beliefs. Third, we did not assess non-binary gender and sexual orientation. However, we have highlighted the importance of technology and online spaces as well as the resistance and negotiations of young Thai female students. Future studies on young female students should be conducted in online spaces and include gender and sexual minority students.

## Data Availability

Data is available upon request from Mahidol University Institutional Review Board (Faculty of Social Sciences and Humanities, Mahidol University Phuttamonthon 4 Rd., Salaya, Phuttamonthon District, Nakhon Pathom 73170. Tel. + 662–441-9180; Fax. + 662–441-9181). In order to protect the privacy of participants, data cannot be deposited to a public repository. While the data has already been stripped of all identifiers (e.g., nicknames, school addresses), they may still be identifiable information since some participants gave significant details about their personal lives, their daily activities, and information about their families. And since sexuality is still a sensitive topic in Thailand, protecting the identities of study participants is very important. For this reason, Mahidol University Institutional Review Board can be contacted to retrieve data from this study.

## Abbreviations

CSE: Comprehensive Sexuality Education
USD: United States Dollars
FGD: Focus Group Discussion
IDI: In-depth Interview
